# Evaluation of acute normovolemic hemodilution in patients undergoing intracranial meningioma resection: A quasi-experimental trial

**DOI:** 10.1097/MD.0000000000008093

**Published:** 2017-09-22

**Authors:** Lei Yang, Hui-Hui Wang, Fu-Sheng Wei, Long-Xian Ma

**Affiliations:** a Department of Gynecology and Obstetrics, The First Affiliated Hospital of Nanchang University, Nanchang; b Department of Anesthesiology, Xinyu People's Hospital, Xinyu; c Department of Anesthesiology, The First Affiliated Hospital of Nanchang University, Nanchang, Jiangxi, P.R. China.

**Keywords:** acute normovolemic hemodilution, blood transfusion, intracranial surgery, meningioma

## Abstract

The aim of this study was to evaluate the safety of acute normovolemic hemodilution (ANH) for patients undergoing intracranial meningioma resection.

Eighty patients (aged 48–65 years) with American Society of Anesthesiologists physical status I–II undergoing intracranial meningioma resection were included in this prospective observational study. The patients were randomly divided into group A (ANH group), which underwent a combination of ANH and intraoperative cell salvage (ICS), and group B (control group), which underwent ICS alone. The study parameters were recorded as baseline values before blood drainage (T_0_), after blood drainage (T_1_), and before (T_2_) and after (T_3_) retransfusion in group A. Whereas in group B, the same parameters were measured 10 minutes after anesthesia induction (T_0_), before surgery (T_1_), and before (T_2_) and after (T_3_) transfusion of autologous blood.

When intraoperative blood loss was <2000 mL, the mean volume of homologous blood transfused in group A patients was 100.8 ± 82.3 mL, compared with the 190.0 ± 91.8 mL in group B. Reduction in homologous blood used in group A was statistically significant (*P* < .05). In group B, 15.1% patients received homologous blood, whereas only 5.9% patients received homologous blood in group A. The difference in heart rate between both groups at different time points was statistically nonsignificant (*P* > .05). The mean hemoglobin and hematocrit levels at T_1_ and T_2_ in group A were lower than in group B (*P* < .05). The prothrombin time and activated partial thromboplastin time in both groups were prolonged significantly after T_2_ (all *P* < .05), but were all within normal range. There were no significant differences in postoperative hospital stay, mortality, and postoperative infection between the 2 groups.

For patients undergoing excision of intracranial meningioma, ANH is an effective procedure to reduce the need for allogeneic transfusions.

## 1 Introduction

Extensive surgical procedures for intracranial meningiomas, especially for deep-seated meningiomas, are often associated with marked blood loss, resulting in the transfusion of allogeneic blood products.^[1]^ Although the potential problems regarding transfusions are well known, transfusion of homologous blood, which increases the risk of infectious diseases and immunological complications, is also a persistent concern.^[2–4]^ To minimize allogeneic blood transfusions, some blood conservation strategies have been introduced and are in routine clinical use. Acute normovolemic hemodilution (ANH) is one such technique that has been used as an alternative to homologous blood transfusions in neurosurgery procedures such as craniosynostosis and spinal procedures.^[5–8]^ ANH with autologous transfusion, which involves the intraoperative removal and storage of whole blood before surgical blood loss, could significantly decrease the requirement for homologous blood during and after surgery.^[9]^ Although a majority of neurosurgical procedures are associated with unwarranted blood loss, there are few studies concerning ANH use in patients undergoing intracranial surgery and a lack of evidence regarding its benefits or its ability to prevent complications.^[7]^ The present study was designed to determine the safety of ANH for patients undergoing intracranial meningioma resection.

## 2 Methods

### 2.1 Study design and participants

The protocol and design of this quasi-experimental trial study was approved by the ethical and scientific committee of The First Affiliated Hospital of Nanchang University, Nanchang, China. Informed consent was obtained from all study participants. Eighty patients (49 men, 31 women, aged 48–65 years, height 160–180 cm, and weight 55–85 kg) with American Society of Anesthesiologists physical status I–II undergoing intracranial meningioma resection were included and randomized into 1 of 2 groups using a simple equal probability randomization scheme. The inclusion criteria were a baseline hemoglobin (Hb) ≥110 g/L, hematocrit (Hct) >35%, platelet ≥100 × 10^9^ L^−1^, normal coagulation system, and no severe pulmonary or cardiovascular diseases. The patients were randomly divided into group A, which received a combination of ANH and intraoperative cell salvage (ICS) (n = 40), and group B, which received ICS alone (n = 40).

### 2.2 Anesthesia and intervention

All patients underwent a standardized general anesthetic technique. Cardiac electrical activity, blood pressure (BP), heart rate (HR), mean arterial pressure (MAP), oxygen saturation, central venous pressure (CVP), end-tidal carbon dioxide (P_ET_CO_2_), and bispectral index were routinely monitored. Anesthesia was induced with an intravenous administration of penehyclidine (0.01 mg/kg), midazolam (0.01 mg/kg), propofol (2 mg/kg), sufentanil (0.5 μg/kg), and rocuronium (0.06 mg/kg). When the trachea was intubated, ventilation was mechanically controlled to maintain P_ET_CO_2_ at 30 to 40 mm Hg. Anesthesia was stabilized with propofol, remifentanil, sufentanil, and cisatracurium. BIS was controlled within a range of 40 to 60.

Patients in group A underwent ANH in which whole blood was collected from the internal jugular vein (20 mL/min) and an equal volume of Gelofusine was transfused. Group B did not receive ANH but underwent basic ICS alone. The target Hct was set at 32% before the surgery. The volume of whole blood collected was about 320 to 670 mL for each patient, which was calculated based on the following formula^[10]^: V = EVB × (Hi-Hf)/Hav; where V = volume of blood collected, EBV = patient's estimated blood volume (70 mL/kg in men and 65 mL/kg in women), Hi = patients initial Hct, Hf = patient's final (desired) Hct after hemodilution (32%), and Hav = average of the initial and final Hct. The collected blood was stored in labeled bags containing acid-citrate-dextrose at room temperature. Retransfusion was started in the ANH group when the main tumor resection of tumor was completed or if Hb was <7 g/dL.

As some studies previously reported,^[11,12]^ Jingjing 3000P device was used for ICS procedure in this study. The standard operative procedures were performed according to the manufacturer's recommended protocols. Trained executive operators performed the blood salvage process. The negative pressure was less than 20 kPa to decrease destruction of red blood cell. When the volume collected was more than 600 to 800 mL, the blood was washed and centrifuged. All salvaged blood would be reinfused into patients.

### 2.3 Parameter measurement

Baseline parameters were measured in all patients, including age, weight, blood loss, and duration of surgery. HR, MAP, CVP, Hb, Hct, prothrombin time (PT), and activated partial thromboplastin time (APTT) were recorded as baseline values before blood drainage (T_0_). These were measured again after blood drainage (T_1_), before retransfusion when the main tumor resection of tumor was completed or if Hb was <7 g/dL (T_2_), and after retransfusion (T_3_) in group A. In group B, the same parameters were measured 10 minutes after anesthesia induction (T_0_), before surgery (T_1_), before transfusion of autologous blood (T_2_), and after transfusion of autologous blood (T_3_). Details regarding postoperative hospital stay, mortality, and postoperative infection were also collected for both groups.

### 2.4 Statistical analysis

Descriptive analyses of variables were used to summarize the data. Normally distributed variables were expressed as mean ± standard deviation and were compared with Student *t* test. The chi-square or Fisher exact test was used to compare proportions between the 2 groups. All reported *P* values were 2 sided, and *P* values < .05 were considered statistically significant. The statistical analysis was performed with the Statistical Package for the Social Sciences version 18.0 software (SPSS Inc, Chicago, IL).

## 3 Results

The patient characteristics are shown in Table 1. There were no significant differences in age, sex, weight, duration of surgery, and intraoperative blood loss between 2 groups (*P* > .05). When intraoperative blood loss was <2000 mL, the mean volume of frozen plasma transfused in group A was 100.8 ± 82.3 mL, compared with the 190.0 ± 91.8 mL in group B. Reduction in homologous blood (red blood cell and frozen plasma) used in group A was statistically significant (*P* < .05). In group B, 15.1% patients received homologous blood, whereas, only 5.9% patients received homologous blood in group A. Six of the patients lost > 2000 mL of blood and could not avoid allogeneic transfusions through ANH (Table 2).

**Table 1:**
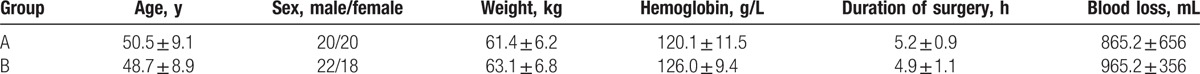
Demographic and clinical characteristics of the 2 groups (mean ± standard deviation).

**Table 2:**
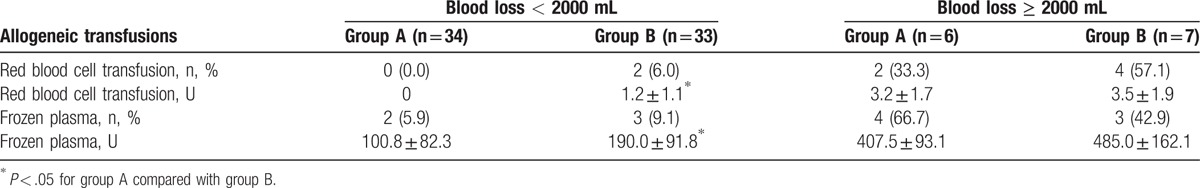
Perioperative allogeneic transfusions.

MAP, HR, and CVP at various time points in both groups are summarized in Table 3. When compared to baseline values (T_0_), a significant reduction in CVP was observed in both groups at T_2_ (*P* < .05). MAP in group A was lower than in group B at T_1_ (*P* < .05). The difference in HR between both groups at the different time points was statistically nonsignificant (*P* > .05). The mean Hb and Hct levels at T_1_ and T_2_ in group A were lower than in group B (*P* < .05); however, the mean Hb and Hct levels at T_1_ – T_3_ were lower than at T_0_ in the 2 groups (*P* < .05) (Table 4). PT and APTT in both groups were prolonged significantly after T_2_ (all *P* < .05), but were all within normal range. PT and APTT at T_3_ were significantly shortened in group A (*P* < .05). There were no significant differences in postoperative hospital stay, mortality, and postoperative infection between the 2 groups (Table 5).

**Table 3:**
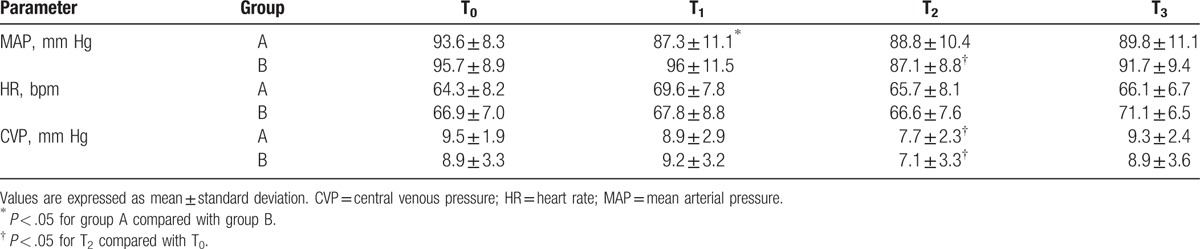
MAP, HR, and CVP at various time points in the 2 groups.

**Table 4:**
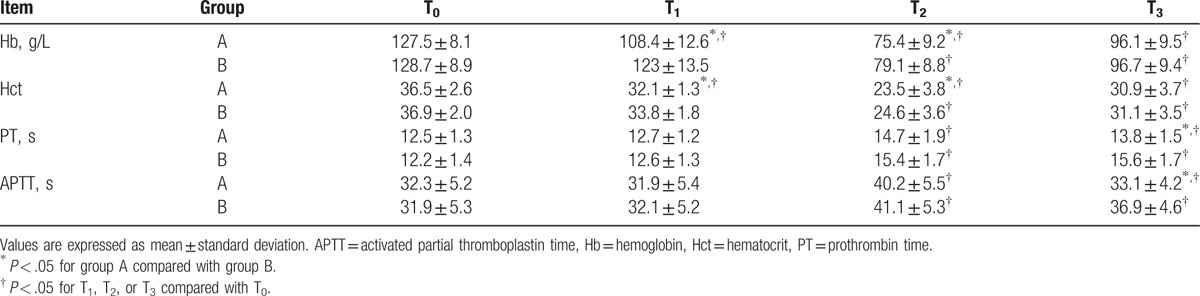
Hb, Hct, PT, and APTT at various time points in the 2 groups.

**Table 5:**
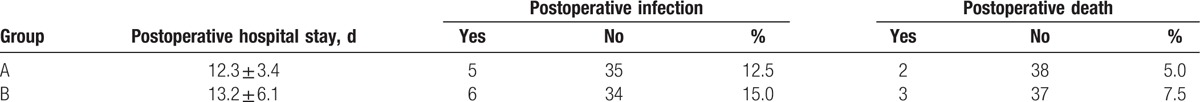
Postoperative hospital stay, infection, and mortality in the 2 groups.

## 4 Discussion

In neurosurgery, during the excision of intracranial meningiomas, the extensiveness of the surgical procedure is considered a major problem and is associated with a significant amount of blood loss due to the high vascular nature of the tumor. Moreover, hemostasis is usually difficult to achieve, especially in deep-seated meningiomas, and blood transfusion is often required. Among the blood conservation strategies currently in use, ANH is a safe technique and is regarded as the most effective for several reasons. First, ANH eliminates inherent delay between donation and operation, and is a cost-effective technique with no administrative or storage costs.^[4]^ Furthermore, ANH refers to no additional personnel costs and no use of expensive equipment being the technique to provide fresh blood for use. Thus, of the alternative methods for blood conservation techniques widely used, ANH is the simplest and most cost effective.^[13,14]^ A lot of research showed that ANH could decrease the requirement for allogeneic blood transfusions.^[15,16]^ The present study also added new evidence that ANH could effectively save the use of homologous blood. Reduction in homologous blood transfusion in the ANH patients was statistically significant.

The majority of studies explaining the mechanisms of ANH involve elevation of cardiac performance and decreasing afterload.^[17]^ Evidence in experimental studies have also suggested that brain and coronary flow increased,^[18]^ although several studies recommended that surgery patients with coronary disease were not suitable for hemodilution. In addition, 1 study has been reported that cardiac output in anesthetized participants with Hct levels of 20% to 25% increased by 16% to 50%.^[19]^ In present study, ANH was performed with a target Hct of 32%. The removal of autologous blood units and hemodilution was accompanied with hemodynamic stability and we did not observe any significant changes in HR.

There is no consensus on the benefits of hemodilution in neurosurgical procedures,^[7,20]^ although it has been reported using in pediatric craniostenosis surgeries.^[21]^ ANH is an alternative technique for obtaining autologous blood that removal of blood from patient, with simultaneous replacement of normovolemia using cell-free fluid. Colloid is the commonly used cell-free fluid, which is still the potential risk factor of hypersensitivity reaction. Our study was performed in adults and analyzed enormous hemodynamic parameters to confirm conclusions for guiding clinical practice. We also found no significant differences in postoperative hospital stay, mortality, and postoperative infection between the 2 groups.

The main limitation of this study is the small sample size. The insufficient sample size and short-term follow-up may limit the significance of the study, and is insufficient to detect small differences in outcome. Furthermore, the volume of ANH may also influence the outcome. Our study failed to assess the association between volume ANH and clinical outcomes. Some studies reported that large volume hemodilution was associated with adverse complications.^[22,23]^ Finally, although the strict exclusion criteria was assumed to minimize biases, the potential bias factors, such as population characteristics, may not be completely eliminated in our study.

## 5 Conclusions

For patients undergoing excision of intracranial meningioma, ANH is an effective procedure to reduce the need for allogeneic transfusions. However, the small sample size precludes final conclusions about the safety for routine use.

## Acknowledgments

We would like to express our gratitude to our coworkers and the patients who participated in this study.
